# Expression of the tumor antigens NY-ESO-1, tyrosinase, MAGE-A3, and TPTE in pediatric and adult melanoma: a retrospective case control study

**DOI:** 10.1007/s00428-024-03846-0

**Published:** 2024-06-18

**Authors:** Stephan Forchhammer, Oltin Tiberiu Pop, Matthias Hahn, Valentin Aebischer, Christian M. Seitz, Christopher Schroeder, Alexandra Liebmann, Michael Abele, Hannah Wild, Ewa Bien, Michal Kunc, Dominik T. Schneider, Katarina Cuk, Isabel Büttel, Carina Flemmig, Magdalena Peters, Mark Laible, Patrick Brück, Özlem Türeci, Ugur Sahin, Lukas Flatz, Ines B. Brecht

**Affiliations:** 1 Department of Dermatology, Eberhard Karls University of Tuebingen, Liebermeisterstrasse 25, 72076 Tuebingen, Germany; 2 Institute for Immunobiology, Kantonsspital St Gallen, St Gallen, Switzerland; 3 Pediatric Hematology and Oncology, Children’s Hospital, Eberhard Karls University of Tuebingen, Tuebingen, Germany; 4 Institute of Medical Genetics and Applied Genomics, Eberhard Karls University of Tuebingen, Tuebingen, Germany; 5 Department of Pediatrics, Hematology and Oncology, Medical University of Gdansk, Gdansk, Poland; 6 Department of Pathomorphology, Medical University of Gdansk, Gdansk, Poland; 7 Clinic of Pediatrics, Dortmund Municipal Hospital, University Witten/Herdecke, Dortmund, Germany; 8 BioNTech SE, Mainz, Germany; 9 Department of Dermatology, Venereology, and Allergology, Cantonal Hospital St. Gallen, St. Gallen, Switzerland

**Keywords:** TAA, Pediatric melanoma, Cancer vaccine, Prognostic factor

## Abstract

Tumor-associated antigens (TAAs) are potential targets for T cell-based immunotherapy approaches in cutaneous melanoma. BNT111, an investigational lipoplex-formulated mRNA-based therapeutic cancer vaccine encoding melanoma TAAs NY-ESO-1, tyrosinase, MAGE-A3, and TPTE, is undergoing clinical testing in adults. Expression of these TAAs in pediatric melanoma is unclear but is a prerequisite for feasibility of this treatment approach in children with melanoma. Our main objective was to characterize expression of those TAAs in pediatric melanomas compared to control cohorts. In this retrospective case control study, protein and transcript expression of NY-ESO-1, tyrosinase, MAGE-A3, and TPTE were analyzed in a cohort of 25 pediatric melanomas, 31 melanomas of young adults, 29 adult melanomas, and 30 benign melanocytic nevi in children using immunohistochemical staining and digital pathology (QuPath) and reverse transcription quantitative PCR. Based on IHC analysis, pediatric melanomas expressed tyrosinase (100.0%), TPTE (44.0%), MAGE-A3 (12.0%), and NY-ESO-1 (8.0%). Young adult melanomas expressed tyrosinase (96.8%), NY-ESO-1 (19.4%), MAGE-A3 (19.4%), and TPTE (3.2%). Adult melanomas expressed tyrosinase (86.2%), MAGE-A3 (75.9%), NY-ESO-1 (48.3%), and TPTE (48.3%). Childhood melanocytic nevi only expressed tyrosinase (93.3%). Expression prevalence of individual TAAs did not differ between subtypes of pediatric melanoma, and no association with prognosis was found. All four TAAs were expressed in pediatric melanoma, albeit NY-ESO-1 and MAGE-A3 to a lesser extent than in adult melanoma. These data support the possibility of investigating vaccines targeting these TAAs for the treatment of pediatric melanoma.

**Supplementary Information:**

The online version contains supplementary material available at 10.1007/s00428-024-03846-0.

## Introduction

Melanoma is responsible for most skin cancer associated deaths worldwide. Despite great progress in the treatment of metastatic melanoma with targeted therapies such as BRAF-inhibitors or immune checkpoint blockade, approximately 55,500 deaths are still caused by melanoma annually [1]. In addition, the incidence of melanoma has increased significantly in recent years and decades [2]. Melanoma is rare in children and adolescents compared to adults. In children under 15 years of age, the incidence is very low at 1.3–1.6/million. However, the incidence of melanoma in adolescents 15–19 years of age is approximately tenfold higher than in children [3, 4]. However, analyses from national and international rare tumor working groups have shown that the incidence of pediatric melanoma may well be underestimated [5, 6]. Although most childhood melanomas are not fatal, there are still cases with an aggressive course [7]. Like in adult melanoma, pediatric melanoma is differentiated into several subtypes, the most prevalent of which are spitzoid melanoma, melanoma arising from a congenital giant nevus, and conventional melanoma, which primarily includes superficial spreading melanoma (SSM), nodular melanoma (NM), and acrolentiginous melanoma (ALM). Patients’ prognoses differ greatly among these subtypes. Children and adolescents with conventional melanomas, those arising from a congenital giant nevus, and rarer melanoma types have poor prognoses similar to adults with melanoma, whereas children with spitzoid melanomas have favorable prognoses [8]. Due to the rarity of the disease, there are no conclusive therapy studies and no standardized therapy recommendations for advanced stage melanoma in children [9].

Therapeutic cancer vaccines targeting tumor-associated antigens (TAAs) could be a promising treatment option for adult melanoma. BNT111 is an investigational intravenously administered vaccine candidate based on a nanoparticulate lipoplex-formulated non-nucleoside-modified uridine-containing mRNA (RNA-LPX) [10]. The BNT111 vaccine encodes four TAAs that are minimally expressed in normal tissue, highly immunogenic, and prevalent in melanoma [10–13]. Of these, New York squamous cell carcinoma of the esophagus 1 (NY-ESO-1), melanoma-associated antigen A3 (MAGE-A3), and transmembrane phosphatase with tensin homology (TPTE) are so-called cancer germline antigens that are not expressed at all in healthy skin tissue and aberrantly activated in melanoma cells [11, 12]. Tyrosinase, in contrast, is a melanocyte differentiation antigen that is physiologically expressed in normal melanocytes, remains expressed upon malignant transformation, and becomes immunogenic [14].

In a phase I trial (Lipo-MERIT, NCT02410733), BNT111 administered alone or combined with PD-1 immune checkpoint inhibitors resulted in a response in adult patients with unresectable melanoma [15]. Such a therapeutic approach may also be effective in children and adolescents; however, no data is available on the expression of these TAAs in pediatric melanoma.

The aim of this retrospective case control study is to characterize the expression of the four BNT111 vaccine-encoded TAAs in pediatric melanomas in comparison to adult melanomas and benign melanocytic nevi of childhood and to evaluate the prognostic value of their expression profile.

## Methods

### Study cohort

The retrospective case control study was performed on formalin-fixed and paraffin-embedded (FFPE) tissue of pediatric melanomas, melanomas of young adults, adult melanomas, and samples from benign melanocytic nevi of childhood. Written informed consent was given by all patients or their legal guardians. Consent was waived for deceased patients or samples older than 5 years. The study was approved by the institutional review board of the University of Tübingen (Project ID: 786/2018BO2). Inclusion criteria for the study were the diagnosis of malignant melanoma between 1 January 2006 and 31 December 2021 and the availability of sufficient FFPE material. Patients who did not have sufficient FFPE material were excluded from the study. The size of the cohorts was based on the availability of FFPE material from pediatric melanomas and melanomas in young adults. The size of the control cohorts was aligned with the size of the study cohort.

The study cohort included 30 patients in childhood (0 < 12 years) and adolescence (12 < 18 years) who were diagnosed with pediatric melanoma as primary tumor (*n* = 28) or metastasis (*n* = 2) at various tumor stages. The control cohorts consisted of 32 young adult melanomas (18–30 years, 32 primary tumors), 30 adult melanomas (over 30 years, 28 primary tumors, two metastasis), and 30 benign melanocytic nevi of childhood (under 18 years). Adult melanomas were matched with the cohort of pediatric melanomas by Breslow tumor thickness (range of 0.1 mm) for primary melanomas and location (lymph nodes, organs, and skin) for metastases. Due to the rarity of the diagnosis in the young adult cohort, tumor parameters could not be matched to the study cohort of pediatric melanomas. Five pediatric melanomas, two young adult melanomas, and one adult melanoma were excluded during the study (see Sample flow, Supplementary Fig. 1). Benign melanocytic nevi of childhood (under 18 years) were selected as a control cohort to compare age-matched non-melanoma patient samples with tumor samples from the test cohort. Pediatric melanoma patients were identified from either the German Registry of Rare Pediatric Tumors (STEP Registry), the Melanoma Registry of the German Dermatological Society in Tübingen, the Archives of Dermatopathology of the University Hospital of Tübingen, or the Archives of Pathology of the University Clinical Center/Medical University of Gdansk. Control patients were identified from the Archives of Dermatopathology of the University Hospital of Tübingen.

### Histologic assessment

The histopathological diagnosis of pediatric melanomas was made by the respective examining dermatopathologist. All samples were further evaluated morphologically and in conjunction with available molecular workups (Comparative Genomic Hybridization (CGH) and Next Generation Sequencing (NGS)) by a reference dermatopathologist from the STEP registry and classified into groups of spitzoid melanoma, conventional melanoma (SSM, NM, ALM, and LMM), melanoma on congenital nevus, other melanoma, and melanoma metastasis. Spitzoid melanomas included all tumors with a spitzoid morphology and no typical driver mutation of conventional melanoma (BRAF, NRAS, KIT, GNAQ, and GNA11). In the case of missing NGS diagnostics, NGS analysis was supplemented in an accredited diagnostic laboratory (Panel containing *BRAF* Exon 11, 15; *NRAS* Exon 2, 3, 4; *KIT* Exon 8, 9, 11, 13, 14, 17, 18; *GNAQ* Exon 5; *GNA11* Exon 5; *CTNNB1* Exon 3; *PDGFRA* Exon 12, 13, 14, 18; *KRAS* Exon 2, 3, 4; *MAP2K1* Exon 2, 3, 6, 7, 11; *TERT*p). Histologic diagnoses of the control cohorts were performed by at least two experienced board-certified dermatopathologists.

### Immunohistochemical staining

FFPE tissue samples were processed using the standard protocols for diagnostic histological examination. Serial sections were cut using a rotary microtome (Microm HM355S, Thermo Fisher Scientific, USA) and placed on poly-L-lysine-coated microscopy slides (catalog number J2800AMNZ, Menzel-Glaser, DE). Single-epitope enzymatic immunohistochemistry (IHC) on FFPE tissue was performed to assess NY-ESO-1, tyrosinase (TYR), MAGE-A3, and TPTE (PTEN2) expression. Morphometric analysis was carried out on whole slide scans acquired with a Pannoramic 250 Flash III digital slide scanner (3D Histech, HU). Quantitative digital morphometry was performed using the QuPath v0.2.3 software for image analysis [16] (Supplementary Fig. 2). The percentage of positive tumor cells was assessed for all markers and tissue samples included. One percent of positively stained tumor cells was chosen as cutoff to determine marker positivity. For details on IHC protocols and digital quantitative assessment, refer to the Supplementary Materials and Methods section.

### RNA expression analysis

Total RNA was extracted from macrodissected FFPE tissue using the bead-based RNA extraction kit RNXtract® (Cerca Biotech GmbH, Germany). Melanin was removed from RNA samples using the OneStep PCR Inhibitor Removal Kit (Zymo Research, DE). *NY-ESO-1*, *Tyrosinase, MAGE-A3*, and *TPTE* mRNA expression were analyzed by reverse transcription quantitative real-time PCR (RT-qPCR) on a CFX96™ qPCR cycler (Bio-Rad, US) using gene-specific oligonucleotides (primer 2 µM, double quenched fluorescent hydrolysis probes 1.2 µM, Integrated DNA Technologies, US, Supplementary Table 1) and OneStep RT-qPCR enzyme mix (UltraPlex 1-Step ToughMix, US). Each RT-qPCR run was validated by a positive control (PC) of in vitro transcription RNA containing all target and reference gene sequences and a no template control (NTC) containing nuclease-free water, both of which needed to meet predefined Cq ranges. Relative gene expression was calculated by normalization of each TAA mean quantification cycle (Cq) against the mean of the three reference genes Pumilio RNA Binding Family Member 1 (*PUM1*), Hypoxanthine Phosphoribosyltransferase 1 (*HPRT1*), and Mitochondrial Ribosomal Protein L19 (*MRPL19*) which had been selected using geNorm (v.3) and NormFinder (v.0953). The delta quantification cycles (dCq) were dichotomized as positive or negative based on previously defined TAA-specific cutoff points which had been selected by measuring normal skin and melanoma tissue samples (Supplementary Fig. 3).

### Statistics

Statistical calculations were performed using IBM SPSS version 26; *p* values < 0.05 were considered statistically significant. Numerical results are reported as means and standard deviations or medians and interquartile ranges (IQR). To analyze the association of protein expression and patient age, logistic regression models and likelihood ratio tests were calculated using Analyse-it (Excel 365 Add-in for Microsoft Excel 5.40). The correlation of IHC and RT-qPCR overall cohorts was determined for each marker by the overall percent agreement (OPA), positive percent agreement (PPA), and negative percent agreement (NPA) rate. Event-free survival rates of TAA positive vs. TAA negative samples were assessed with Kaplan–Meier curves and compared using the log-rank test. In the case of primary melanoma analysis, an event was defined as the first occurrence of metastasis or death by melanoma; in the case of metastasis analysis, an event was defined as the first occurrence of further metastasis or death by melanoma.

## Results

### Epidemiological data

Samples from 115 patients were examined for expression of NY-ESO-1, tyrosinase, MAGE-A3, and TPTE. Pediatric melanomas (*n* = 25, 12:13 male:female, ages 1–17 years, median tumor thickness 3.25 mm) were compared with three control cohorts: melanomas in young adults (*n* = 31, 13:18 male:female, ages 20–30 years, median tumor thickness 0.75 mm), adult melanomas (*n* = 29, 13:16 male:female, ages 33–95 years, median tumor thickness 2.5 mm), and benign melanocytic nevi of childhood (*n* = 30, 15:15 male:female, ages 0–17 years). Pediatric melanomas were predominantly stage II (48%) at diagnosis according to AJCC 2017, while melanomas in young adults were predominantly stage I (77%), and adult melanomas were evenly distributed between stages I, II, and III (each 31%). The most common subtype of pediatric melanomas was spitzoid melanoma (48%), whereas superficial spreading melanoma was predominant in young adult (71%) and adult melanomas (66%) (Table 1).

**Table 1:** Epidemiological data

	Pediatric melanoma (age < 18) *n* = 25	Melanoma in young adults (age 18–30) *n* = 31	Melanoma in adults (age > 30) *n* = 29	Benign nevi of childhood (age < 18) *n* = 30
Age at diagnosis (years)				
Min./Max	1/17	20/30	33/95	0/17
Median (+ IQR)	10 (6.3/13.0)	26 (23.3/28.0)	68 (47.0/76.3)	12 (6.0/14.0)
Mean value (± SD)	9.5 (± 4.5)	25.7 (± 3.2)	64.2 (± 17.6)	9.8 (± 5.3)
Sex (*n*, %)				
Male (*n*, %)	12 (48%)	13 (42%)	13 (45%)	15 (50%)
Female (*n*, %)	13 (52%)	18 (58%)	16 (55%)	15 (50%)
Primary tumor				
Tumor thickness (Breslow, mm), Median (+ IQR)	3.25 (2.125/5.000)	0.75 (0.450/1.895)	2.5 (1.533/4.458)	na
Ulceration (*n*, %)	4 (16%)	1 (3%)	9 (31%)	na
Histologic subtype				
Superficial spreading melanoma (SSM) (*n*, %)	4 (16%)	22 (71%)	19 (66%)	na
Nodular melanoma (NM) (*n*, %)	1 (4%)	3 (10%)	2 (7%)	na
Lentigo maligna melanoma (LMM) (*n*, %)	0 (0%)	0 (0%)	2 (7%)	na
Acral lentiginous melanoma (ALM) (*n*, %)	1 (4%)	2 (6%)	2 (7%)	na
Spitzoid melanoma (*n*, %)	12 (48%)	4 (13%)	1 (3%)	na
Melanoma on congenital nevus (*n*, %)	3 (12%)	0 (0%)	0 (0%)	na
Metastasis (*n*, %)	2 (8%)	0 (0%)	2 (7%)	na
Others (*n*, %)	2 (8%)	0 (0%)	1 (3%)	na
Tumor stage at diagnosis (AJCC 2017)				
I	5 (20%)	24 (77%)	9 (31%)	na
II	12 (48%)	5 (16%)	9 (31%)	na
III	5 (20%)	2 (7%)	9 (31%)	na
IV	2 (8%)	0 (0%)	2 (7%)	na
n.a	1 (4%)	0 (0%)	0 (0%)	na
Localization				
Head/neck (*n*, %)	5 (20%)	6 (19%)	7 (24%)	12 (40%)
Trunk (*n*, %)	5 (20%)	12 (39%)	13 (45%)	7 (23%)
Upper extremities (*n*, %)	8 (32%)	4 (13%)	3 (10%)	3 (10%)
Lower extremities (*n*, %)	6 (24%)	9 (29%)	6 (21%)	8 (27%)
Others/unknown (*n*, %)	1 (4%)	0 (0%)	0 (0%)	0 (0%)

### Expression profile of TAAs in pediatric melanoma, melanoma in young adults, adult melanoma, and benign melanocytic nevi of childhood

**Fig. 1:**
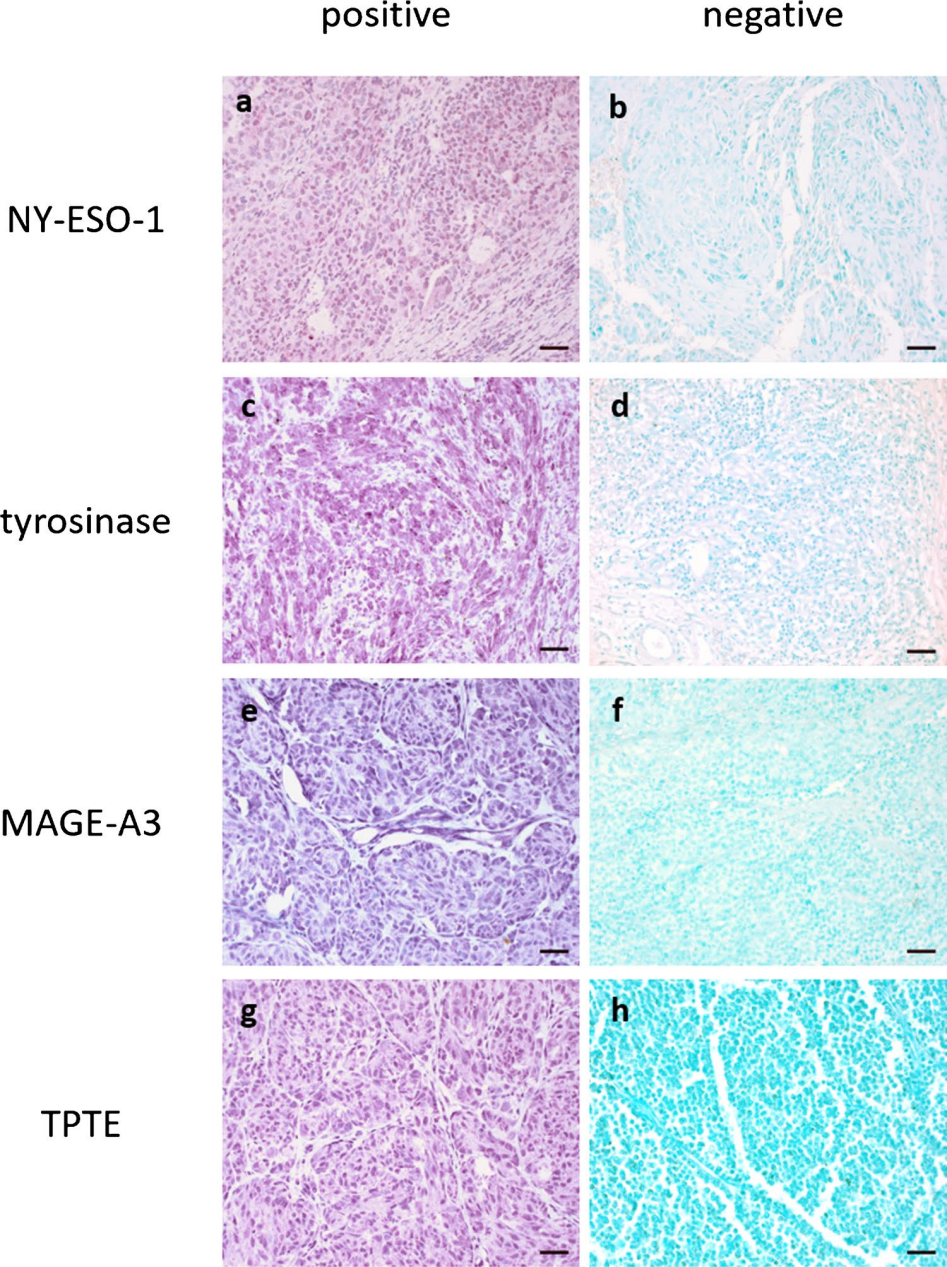
Immunohistochemical staining of TAA protein expressions in pediatric melanomas. **a** Positive NY-ESO-1 staining of patient K5, **b** negative NY-ESO-1 staining of patient K1, **c** positive tyrosinase staining of patient K22,** d** negative tyrosinase staining of adult patient A20 as negative staining of tyrosinase is absent in our cohort of pediatric melanoma,** e** positive MAGE-A3 staining of patient K1,** f** negative MAGE-A3 staining of patient K5,** g** positive TPTE staining of patient K1,** h** negative TPTE staining of patient K27. Scale bars are equal to 50 µm

**Fig. 2:**
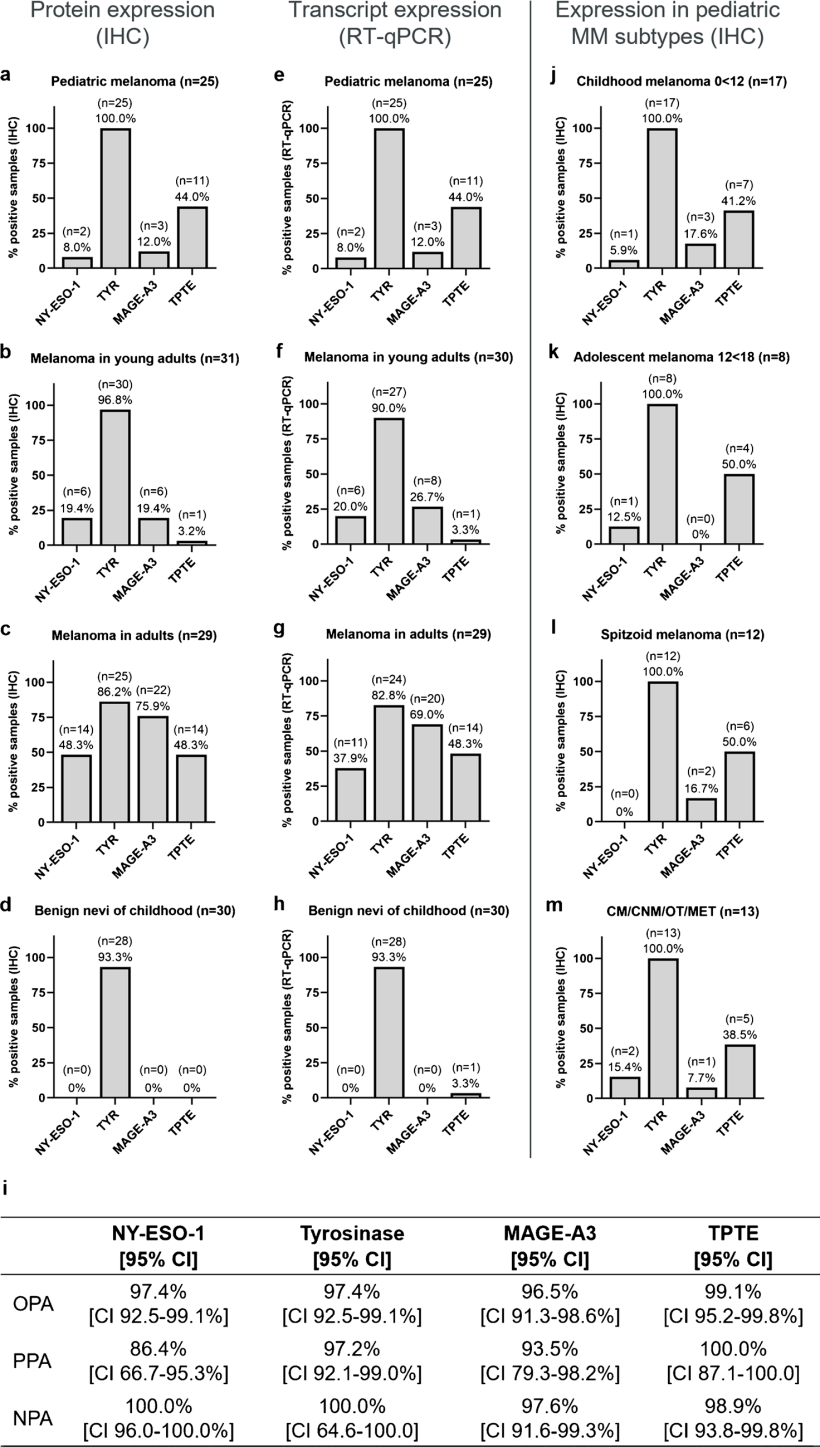
Expression levels of NY-ESO-1, tyrosinase, MAGE-A3, and TPTE** a–d** Protein expression by immunohistochemistry. **a** Pediatric melanoma, **b** melanoma in young adults, **c** adult melanoma, **d** benign nevi of childhood. **e–h** Transcript expression by RT-qPCR**. e** pediatric melanoma, **f** melanoma in young adults, **g** adult melanoma, **h** benign nevi of childhood. Samples with late or no Cqs for targets were classified as negative. **i** Concordance of IHC and RT-qPCR over all samples (*n* = 114)**.** OPA overall percent agreement, PPA positive percent agreement, NPA negative percent agreement. **j–m** Protein expression of TAAs in different age strata and subtypes of pediatric melanoma. **j** Childhood melanoma defined as patients under 12 years, **k** adolescent melanoma defined as patients from 12 to 18 years, **l** Spitzoid melanoma, **m** non-spitzoid melanoma. CM conventional melanoma (includes SSM, NM, and ALM), CNM congenital nevus melanoma, MET metastasis, OT other type of melanoma

TAA expression in primary tumor and metastasis samples was analyzed on the protein level by immunohistochemical staining and quantified automatically using QuPath (Fig. 1). Cutoff for positivity for all markers was defined as ≥ 1% positively stained tumor cells. The expression pattern of the four markers showed great variability in terms of homogeneity within the same study group as well as between different study groups with a tendency towards a more homogeneous staining for tyrosinase (Supplementary Fig. 4). Staining intensity was not assessed in this study because of non-standardized preanalytical conditions, such as cold ischemia time, fixation time, and fixative. In general, tyrosinase was frequently expressed in all four cohorts (86.2–100.0%), while NY-ESO-1, MAGE-A3, and TPTE were less frequently expressed overall with marked differences between cohorts (Fig. 2a–d). Supplementary Tables 2 to 5 list TAA expression for each cohort in relation to clinical data, follow-up, and outcome.

Tyrosinase was expressed in all pediatric melanoma samples, followed in frequency by TPTE (44.0%, *n* = 11), MAGE-A3 (12.0%, *n* = 3), and NY-ESO-1 (8.0%, *n* = 2) (Fig. 2a, Supplementary Table 2). Tyrosinase was the only TAA expressed in a majority (56.0%, *n* = 14) of pediatric melanoma samples. Some pediatric melanomas expressed two (24.0%, *n* = 6) or three (20.0%, *n* = 5) TAAs, and none expressed all four.

Almost all young adult melanomas expressed tyrosinase (96.8%, *n* = 30) and expressed the other three TAAs in notably different frequencies than pediatric melanomas (Fig. 2b, Supplementary Table 3). TPTE was much less frequently expressed in young adult melanomas (3.2%, *n* = 1), whereas NY-ESO-1 and MAGE-A3 were more frequently expressed (both 19.4%, *n* = 6). Most young adult melanomas expressed one TAA (61.3%, *n* = 19), almost a third expressed two TAAs (29.0%, *n* = 9), while few expressed three TAAs (6.5%, *n* = 2). Simultaneous expression of all four TAAs was not detected in young adult melanomas.

Adult melanomas expressed NY-ESO-1 (48.3%, *n* = 14) and MAGE-A3 (75.9%, *n* = 22) much more frequently than either young adult or pediatric melanomas (Fig. 2c, Supplementary Table 4). Adult melanomas expressed TPTE (48.3%, *n* = 14) as frequently as pediatric melanomas. Again, adult melanomas frequently expressed tyrosinase, though slightly less so than the other melanoma cohorts (86.2%, *n* = 25). In contrast to the younger melanoma cohorts, a minority of adult melanomas expressed one TAA (20.7%, *n* = 6), with most expressing either two (13.8%, *n* = 4), three (37.9%, *n* = 11), or four (24.1%, *n* = 7) TAAs. One sample expressed none of the TAAs (3.4%).

Almost all benign melanocytic nevi of childhood controls except for two expressed tyrosinase (93.3%, *n* = 28), but none of the other showed detectable TAA expression (Fig. 2d, Supplementary Table 5).

We also investigated transcript expression of the TAAs in our cohorts using RT-qPCR (Fig. 2e–h). RT-qPCR and IHC results agreed well for all four TAAs over all four cohorts, with OPA ranging from 96.5 to 99.1% (Fig. 2i). Ten samples (*n*[NY-ESO-1] = 3, *n*[tyrosinase] = 3, *n*[MAGE-A3] = 4, *n*[TPTE] = 1) showed inconsistent transcript and protein expression data. One of these samples resulted in two inconsistent cases; the other cases were distributed to different samples. In eight of eleven inconsistent cases the transcript result was negative with late or undetected targets, only for one case the dCq result was located near its RT-qPCR cutoff. These samples predominantly represented junctional tumors, thin tumors, had few tumor cells, or were archived for extended times.

To further explore differences in TAA expression frequency within the cohort of pediatric melanomas, TAA expression was analyzed separately for children (*n* = 17, ages 0 < 12 years) and adolescents (*n* = 8, ages 12 < 18 years) (Fig. 2j, k). The only marked difference between these subgroups was found in MAGE-A3 expression which should not be overinterpreted due to the limited sample number.

To investigate whether TAA expression differed between melanoma subtypes with different prognostic outcomes, we compared the expression patterns of spitzoid melanomas, which have a favorable prognosis, to the combined expression patterns of conventional melanoma (CM; including SSM, NM, and ALM), congenital nevus melanoma (CNM), other subtypes (OT), and metastatic melanoma (MET), which all have a poor prognosis (Fig. 2l, m). All NY-ESO-1-positive samples fell within the CM/CNM/OT/MET group. Additionally, MAGE-A3 and TPTE expression frequency was marginally lower in the CM/CNM/OT/MET group.

### Expression of TAAs in relation to patient age

**Fig. 3:**
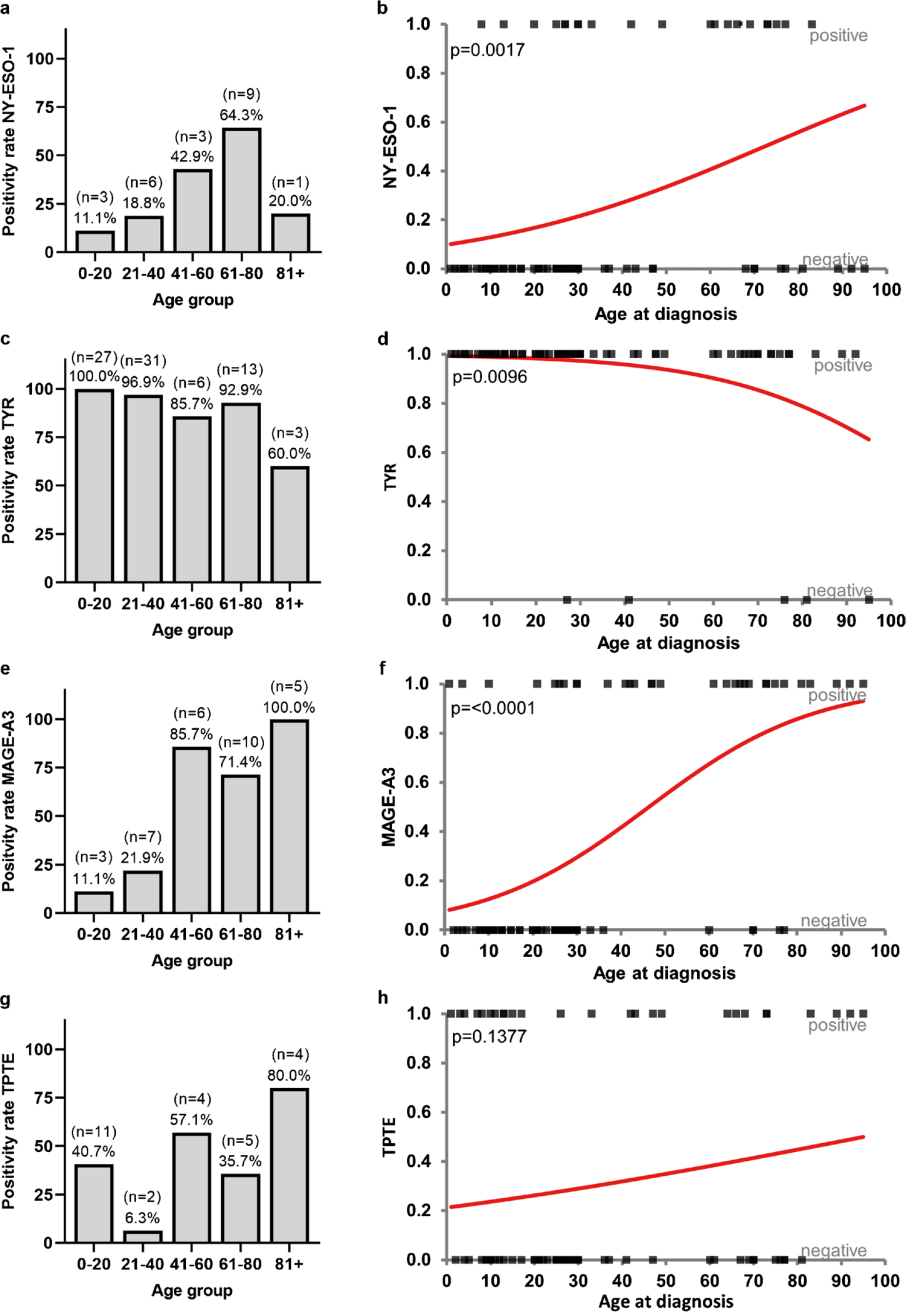
Expression of TAAs (IHC) in relation to patient age Samples were combined in age groups of 20 years: 0–20 (*n* = 27), 21–40 (*n* = 32), 41–60 (*n* = 7), 61–80 (*n* = 14), 81 + (*n* = 5). **a, b** NY-ESO-1, **c, d** tyrosinase, **e, f** MAGE-A3, **g, h** TPTE, **a, c, e, g** relative expression frequency per age group in pediatric, young adult, and primary adult melanomas, **b**, **d**, **f**, **h** logistic regression models over pediatric, young adult, and adult melanoma samples. *p* Values were determined with likelihood ratio G^2^ test

To assess whether the differences of TAA expression patterns in different age strata were associated with patient age, we plotted TAA expression frequencies versus age (20-year bins) (Fig. 3a, c, e, g) and performed logistic regression models (Fig. 3b, d, f, h). The analysis was performed over all melanoma samples including metastatic samples. The first group (0–20 years) contains all pediatric (*n* = 25) and young adult samples until 20 years (*n* = 2, *n*_total_ = 27). The age groups 41–60 and 80 + are underrepresented with seven and five samples, respectively.

Regression analysis showed that as patient age increased, the prevalence of tyrosinase expression decreased significantly while NY-ESO-1 and MAGE-A3 expression prevalence increased significantly. The slight decrease in NY-ESO-1 expression for patients older than 80 years is negligible because of the small patient number in that age group (Fig. 3a, b). Prevalence of TPTE expression did not significantly correlate with patient age (Fig. 3g, h).

### Expression of TAAs in relation to patient survival

**Fig. 4:**
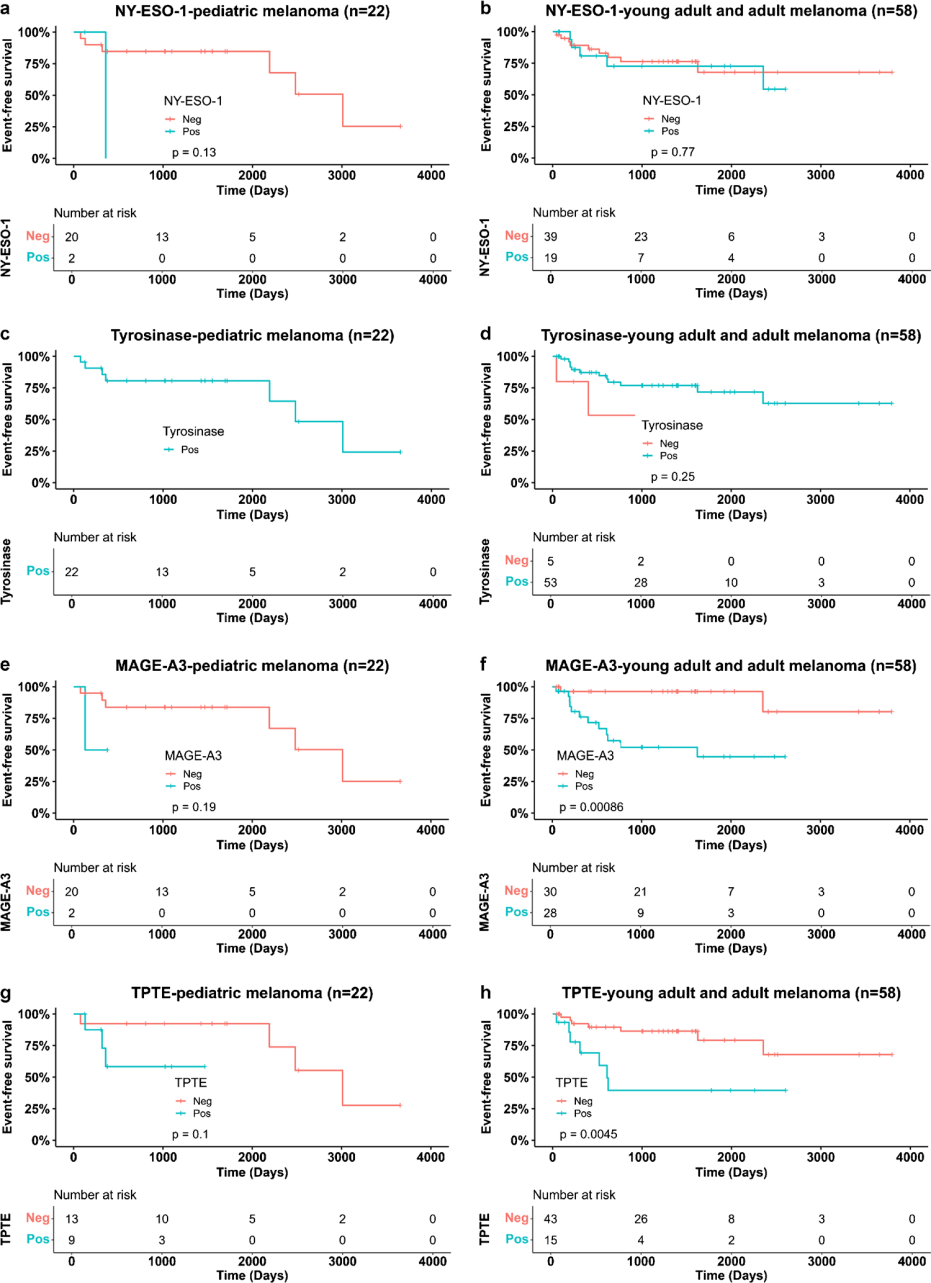
Event-free survival of melanoma patients stratified by TAA expression **a, c, e, g** Pediatric melanoma. As all pediatric melanomas were positive for tyrosine (**c**), an analysis was not possible. **b, d, f, h** Non-pediatric melanoma. **a, b** NY-ESO-1 expression; **c, d** tyrosinase expression; **e, f** MAGE-A3 expression; **g, h** TPTE expression

To investigate whether expression of individual TAAs may have a prognostic value for pediatric melanoma patients, we performed Kaplan–Meier analyses of event-free survival in a cohort of 22 patients with available follow-up data, four of whom died during the analyzed period (Fig. 4). Statistical analysis was not performed for tyrosinase expression because this TAA was detected in all pediatric melanoma samples (Fig. 4c). No prognostic significance was found for the other three TAAs (NY-ESO-1, *p* = 0.13; MAGE-A3, *p* = 0.19; TPTE, *p* = 0.1; Fig. 4a, e, g). These results are also evident in deceased pediatric melanoma patients. Three of the four deceased children expressed only tyrosinase. Only one deceased patient expressed three TAAs (NY-ESO-1, tyrosinase, and TPTE) (see Supplementary Table 2). In contrast, a comparison of the NGS analyses shows that two of the four deceased patients had a *BRAF* V600 mutation and one patient had an *NRAS* mutation (see Supplementary Table 2).

For comparison, we performed the same survival analysis on a combined cohort of 58 young adults and adults with melanoma with available follow-up data, seven of whom died during the analyzed time period. Both MAGE-A3 (*p* < 0.001; Fig. 4f) and TPTE (*p* = 0.0045; Fig. 4h) expression were significantly associated with melanoma-related events, whereas NY-ESO-1 (*p* = 0.77; Fig. 4b) and tyrosinase (*p* = 0.25; Fig. 4d) expression were not significantly associated with prognosis. Six of the seven adults in the cohort who died of melanoma showed expression of more than one TAA. Notably, expression of MAGE-A3 was detected in all seven deceased patients in this cohort (Supplementary Table 3 and 4).

## Discussion

In our analysis of pediatric melanoma samples, we made several key findings regarding the expression of NY-ESO-1, tyrosinase, MAGE-A3, and TPTE.

First, as shown by IHC, robust protein expression of all four TAAs was detected in our cohort of pediatric melanomas. The expression profiles were similar in children and adolescents and differed only slightly between spitzoid melanomas and melanoma subtypes with poor prognoses. Many pediatric melanomas belong to the group of spitzoid melanomas which have favorable prognosis. Few treatment options are available for conventional melanomas with poor outcomes [7, 8]. The expression of the four TAAs encoded by BNT111 in our pediatric cohort supports the possibility of investigating vaccines targeting these TAAs for the treatment of pediatric melanoma.

The expression profiles of TAAs in the control cohorts of adult melanomas were similar to previously published data. In particular, the expression of NY-ESO-1, tyrosinase, and MAGE-A3 has been well studied. Studies have found that 0–45% of adult primary melanomas and 14–62% of metastatic melanomas express NY-ESO-1 (48.3% primary and metastatic melanomas in this study) [17–19], 61–100% of adult melanomas express tyrosinase (86.2% in this study) [19–21], and 15–37% of adult primary melanomas and 25–81% of metastatic melanomas express MAGE-A3 (75.9% primary and metastatic melanomas in this study) [18, 22, 23]. However, MAGE-A3 expression in our control cohort of adult melanomas (75.9%) was higher than expected from previous study results when considering our cohort’s high proportion of primary melanomas (27 of 29 samples). In line with previous data, the control cohort of benign melanocytic nevi in childhood showed exclusive expression of tyrosinase [24]. The expression of TPTE has not been well studied in melanoma. TPTE is a germline-specific protein that is abnormally transcribed in cancers such as liver, prostate, and lung cancer [11, 25–27]. Our study highlights the expression profile of TPTE in pediatric and adult melanomas and describes TPTE as the second most frequently expressed TAA in pediatric melanomas after tyrosinase.

Second, our data suggests a correlation of NY-ESO-1, tyrosinase, and MAGE-A3 expression in melanomas with patient age. Tyrosinase expression rate decreased significantly with patient age, while NY-ESO-1 and MAGE-A3 expression rates increased significantly. Increasing rates of MAGE-A3 expression with age have been described in esophageal carcinoma [28]. In contrast to our results, a study on triple negative breast carcinoma described a decrease in NY-ESO-1 expression with age [29], and a study of melanoma failed to find a correlation between NY-ESO-1 expression and age [17].

Third, our data could not establish prognostic value of individual TAA expression in our cohort of pediatric melanoma patients with available follow-up data, potentially due to the low number of cases and tumor-specific events. However, mutational analysis of pediatric melanoma samples using NGS is striking. Three out of four patients who died from melanoma had either *BRAF* V600 or *NRAS* mutations (Supplementary Table 2). These results are in line with studies showing a different genetic background for spitzoid melanomas and melanomas with worse prognoses [7, 8, 30]. Our results do show significant associations of MAGE-A3 and TPTE expression with melanoma-related events in young adult and adult melanoma patients, supporting existing evidence for MAGE-A3’s prognostic value and introducing TPTE as a possible prognostic indicator [31, 32], highlighting the need for these and other TAAs to be investigated in larger study populations.

Limitations of our study are that the pediatric and young adult melanoma sample selection was restricted by the rarity of the disease in these age groups and the limited availability of FFPE tumor material. As a result, no exact matching was possible between the study and control cohorts. Therefore, the young adult cohort comprised thinner melanomas than the pediatric cohort and exhibited a lower rate of ulceration and melanoma-related events compared to the group of adult melanomas. Since an influence of tumor thickness on the expression of the investigated TAAs cannot be reliably excluded, the comparability between pediatric melanomas and adult melanomas to the group of melanomas in young adults may be limited. In addition, due to the limited number of cases, the reliability of the statistical analyses is limited, especially in the subgroup analyses. Most of the follow-up data were censored because of the early follow-up timepoints, for a few cases follow-up data were absent, as samples were collected from many locations, and others were stored for long durations.

In summary, our study describes the expression of the TAAs NY-ESO-1, tyrosinase, MAGE-A3, and TPTE in pediatric melanoma and adult control cohorts as well as benign nevi, on transcript and on protein level, and addresses one of the prerequisites to consider extension of BNT111 cancer vaccine clinical testing to the pediatric population.

### Supplementary Information

Below is the link to the electronic supplementary material.

Supplementary file1 (DOCX 1975 KB)

## Data Availability

Data generated or analyzed during this study are included in this published article and its supplementary information files.
