# Supplementary material for: Expression of the tumor antigens NY-ESO-1, tyrosinase, MAGE-A3, and TPTE in pediatric and adult melanoma: a retrospective case control study
Source: Virchows Arch. 2024 Jun 18;485(2):335–46. doi: 10.1007/s00428-024-03846-0 (PMC11329550; doi:10.1007/s00428-024-03846-0)
Supplement: Supplementary file 1 — Supplementary file1 (DOCX 1975 KB) [file 428_2024_3846_MOESM1_ESM.docx]

Supplementary information

# Supplementary Materials and Methods

*Immunohistochemical staining*

IHC protocols for NY-ESO-1, TYR, MAGE-A3, and TPTE were optimized by performing the respective single-epitope enzymatic immunostains on tissue sections from FFPE positive control tissues known to express these markers: human testis for NY-ESO-1, MAGE-A3, and TPTE, and human skin for TYR. Each protocol followed the manufacturer`s instructions with regards to antigen retrieval methods and antibody concentrations, and included both positive and negative controls for the primary antibody. Two slides from the corresponding positive control tissues were included with each batch of test tissues and used as positive and negative controls for the primary antibodies.

**TYR IHC:** The slides were dewaxed in xylene, rehydrated, subjected to heat-induced epitope retrieval (HIER) in a microwave oven using a Tris-EDTA target retrieval solution (pH 9) for 40 minutes, and allowed to cool to room temperature. Endogenous horseradish peroxidase activity was blocked using 3% H_2_O_2_ (catalog number 160-0-029, Laboratorium Dr. G. Bichsel AG, CH) for 15 minutes at room temperature, followed by a streptavidin and a biotin blocking step (15 minutes each at room temperature) (catalog number SP-2002, Vector Laboratories, CA, USA), a 30-minute incubation with 3% skim milk at room temperature, and an overnight incubation at 4°C with a mouse anti-human TYR primary antibody (catalog number M3623, Dako, DK; dilution 1:50). Slides were then incubated for 1 hour at room temperature with a biotinylated anti-mouse secondary antibody (1:200), and then for another hour at room temperature with HRP-labeled streptavidin (1:200) (catalog number D31-1, GBI Labs, UK). The signal was visualized using the Vector VIP kit (violet chromogen) (catalog number SK-4600, Vector Laboratories, CA, USA). The slides were dehydrated with alcohol, cleared with xylene and mounted with Pertex (catalog number 41-4011-00, Medite, DE) after methyl green counterstaining (catalog number H-3402, Vector Laboratories, CA, USA).

**NY-ESO-1 IHC:** The slides were dewaxed in xylene, rehydrated, subjected to HIER in a microwave oven using a Tris-EDTA target retrieval solution (pH 9) for 20 minutes, and allowed to cool to room temperature. Endogenous horseradish peroxidase activity was blocked using 3% H2O2 (catalog number 160-0-029, Laboratorium Dr. G. Bichsel AG, CH) for 15 minutes at room temperature, followed by a 30-minute incubation with 3% skim milk at room temperature, and an overnight incubation at 4°C with a mouse anti-human NY-ESO-1 primary antibody (catalog number ab242416, Abcam, UK; dilution 1:200). Slides were then incubated for 1 hour at room temperature with an HRP-labeled donkey anti-mouse secondary antibody (catalog number 715-035-151, Jackson Immunoresearch, PA, USA; dilution 1:400). The signal was visualized using the Vector VIP kit (violet chromogen) (catalog number SK-4600, Vector Laboratories, CA, USA). The slides were dehydrated with alcohol, cleared with xylene and mounted with Pertex (catalog number 41-4011-00, Medite, DE) after methyl green counterstaining (catalog number H-3402, Vector Laboratories, CA, USA).

**MAGE-A3 IHC:** The slides were dewaxed in xylene, rehydrated, subjected to heat-induced epitope retrieval (HIER) in a microwave oven using a Tris-EDTA target retrieval solution (pH 9) for 20 minutes, and allowed to cool to room temperature. Endogenous horseradish peroxidase activity was blocked using 3% H2O2 (catalog number 160-0-029, Laboratorium Dr. G. Bichsel AG, CH) for 15 minutes at room temperature, followed by a 30-minute incubation with 3% skim milk at room temperature, and an overnight incubation at 4°C with a rabbit anti-human MAGE-A3 primary antibody (catalog number ab223162, Abcam, UK; dilution 1:500). Slides were then incubated for 1 hour at room temperature with an HRP-labelled donkey anti-rabbit secondary antibody (catalog number 711-035-152, Jackson Immunoresearch, PA, USA; dilution 1:200). The signal was visualized using the Vector VIP kit (violet chromogen) (catalog number SK-4600, Vector Laboratories, CA, USA). The slides were dehydrated with alcohol, cleared with xylene and mounted with Pertex (catalog number 41-4011-00, Medite, DE) after methyl green counterstaining (catalog number H-3402, Vector Laboratories, CA, USA).

**TPTE (PTEN2) IHC:** The slides were dewaxed in xylene, rehydrated, subjected to heat-induced epitope retrieval (HIER) in a microwave oven using a Tris-EDTA target retrieval solution (pH 9) for 20 minutes, and allowed to cool to room temperature. Endogenous horseradish peroxidase activity was blocked using 3% H2O2 (catalog number 160-0-029, Laboratorium Dr. G. Bichsel AG, CH) for 15 minutes at room temperature, followed by a 30-minute incubation with 3% skim milk at room temperature, and an overnight incubation at 4°C with a rabbit anti-human PTEN2 primary antibody (catalog number PA5-62027, Invitrogen, MA, USA; dilution 1:100). Slides were then incubated for 1 hour at room temperature with an HRP-labeled donkey anti-rabbit secondary antibody (catalog number 711-035-152, Jackson Immunoresearch, PA, USA; dilution 1:200). The signal was visualized using the Vector VIP kit (violet chromogen) (catalog number SK-4600, Vector Laboratories, CA, USA). The slides were dehydrated with alcohol, cleared with xylene and mounted with Pertex (catalog number 41-4011-00, Medite, DE) after methyl green counterstaining (catalog number H-3402, Vector Laboratories, CA, USA).

*Image analysis*

Quantitative morphometry was performed on all stained slides using the QuPath v0.2.3 software platform for whole slide image analysis (Edinburgh, UK) [16] (Supplementary Figure 2). Whole slide scans were individually loaded into the software. For each slide image stain vector (i.e., color) and background estimates were applied to improve stain separation within QuPath by selecting a representative area containing background along with examples of strong nuclear counterstaining and chromogen staining. QuPath’s “Estimate stain vectors” command was applied to identify stain vectors within this region. The “Positive Cell Detection” command was then used to identify cells across the selected region of interest based upon nuclear staining. The full extent of each cell was estimated based upon a constrained expansion of the nucleus region, and up to 33 measurements of intensity and morphology, including nucleus area, circularity, staining intensity for counterstaining and chromogen, and nucleus/cell area ratio were calculated. A random trees classifier (RTrees) was then interactively trained to distinguish tumor cells from all other detections (comprising non-tumor epithelial cells, immune cells, stromal cells, necrosis, or any artefacts misidentified as cells). Cells were classified as positive or negative based upon a single intensity threshold applied to the maximum optical density of the detection chromogen within the nucleus or cytoplasm of the cell depending on the expression pattern. In the case of NY-ESO-1 and tyrosinase, positive cells were considered those showing cytoplasmic expression, while in the case of MAGE-A3, and TPTE, a cell was considered positive if nuclear and/or cytoplasmic expression was observed.

Summary scores were generated as the percentage of cells classified as positive, with “other” detections removed.

# Supplementary Figures


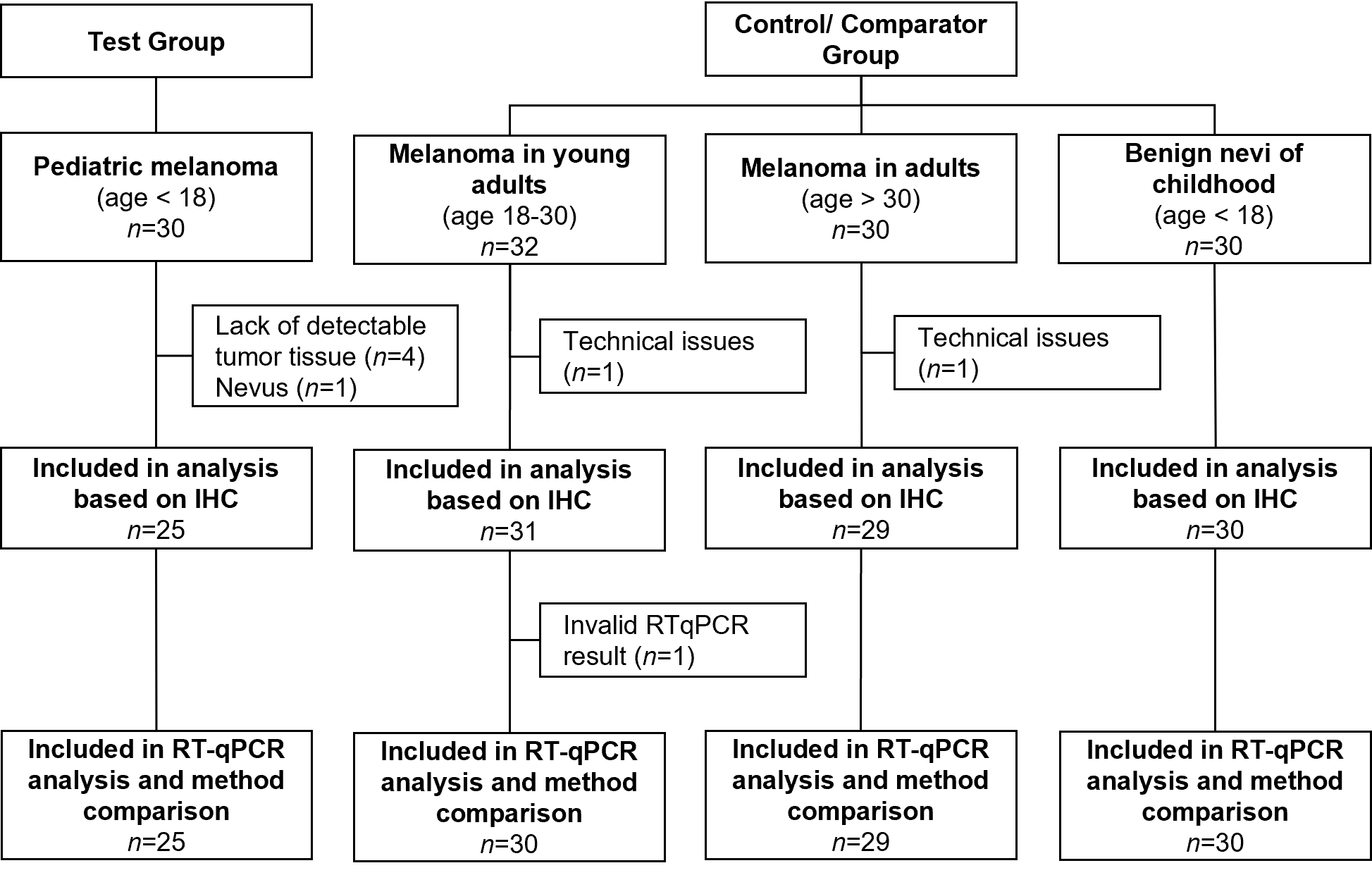


**Supplementary Figure 1 Sample Flow** In the test cohort of pediatric melanoma, 30 patients were recruited. 5 patients were not included in the analysis (4 patients with non-sufficient tumor tissue, 1 patient classified as nevus in the pathological review). In the control group of young adult patients with melanoma, 32 patients were recruited, one patient was excluded due to technical problems in the workup. In the control group of adult patients over 30 years of age, 30 patients were recruited and matched with the 30 recruited patients of the pediatric melanoma cohort (matched by tumor thickness according to Breslow). One patient had to be excluded from this group due to technical problems in the workup. The control group of benign nevi of childhood consisted of 30 patients who were all included in the analysis.


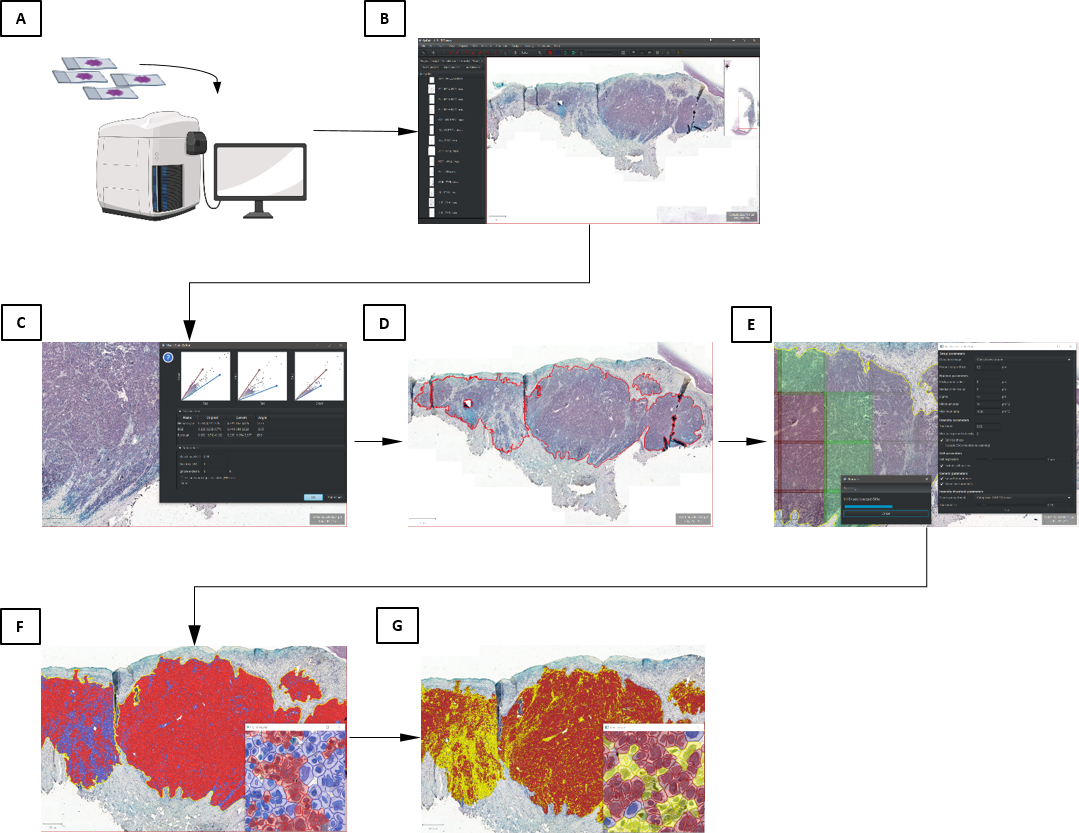


Supplementary Figure 2 Workflow for digital image analysis using the QuPath software platform a Scanning of the slides with tissue sections stained e.g. for tyrosinase (TYR) using enzymatic immunohistochemistry. b Whole slide scans (WSS) are uploaded on the QuPath software platform in a project file. c In a first step, WSS are designated as «Brightfield (H-DAB)» type, and stain vectors are estimated using the Visual Stain Editor. d The region of interest is marked for downstream analysis using the Wand Tool (yellow contour). e The Positive Cell Detection tool is used to assess the number and percentage of TYR-positive cells using Methyl Green as counterstaining and a single intensity threshold for TYR. f Overlay of cell detection mask (positive cells – red mask; negative cells – blue mask). g The Train Object Classifier allows for cell classification into TYR-positive tumor cells (red mask), TYR-negative tumor cells (blue mask), and non-tumor cells (yellow mask).


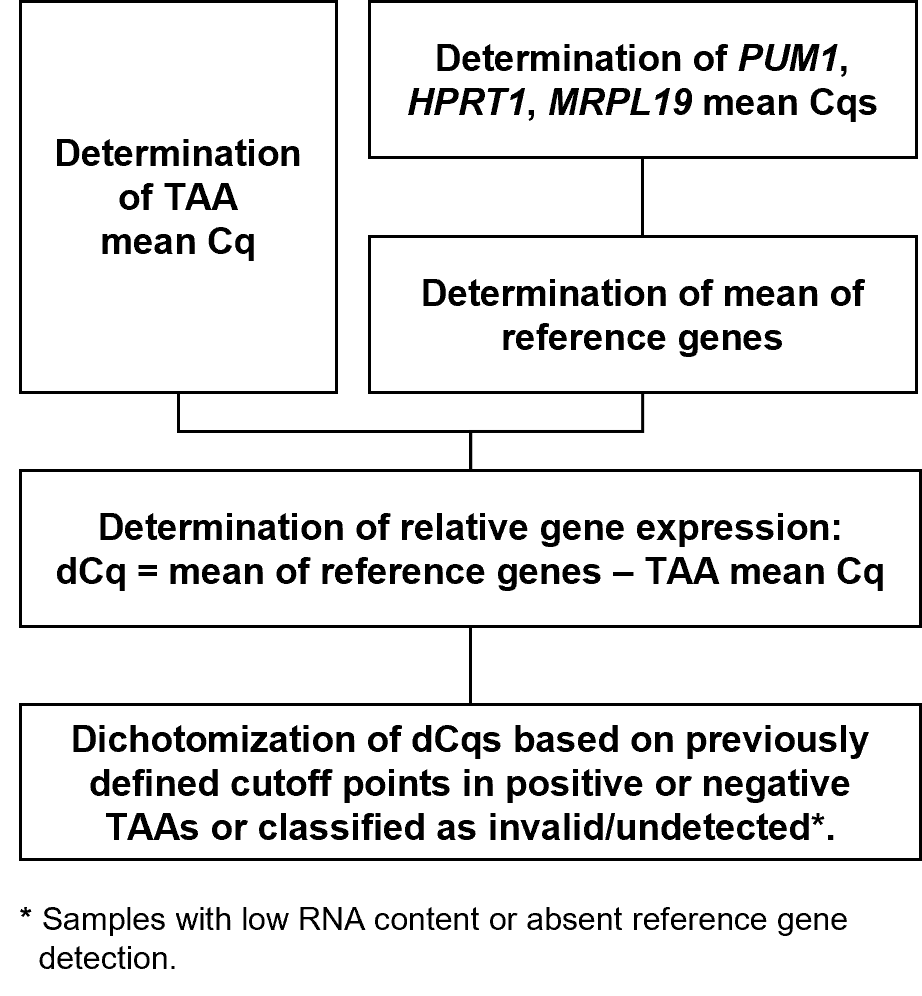


Supplementary Figure 3 Dataflow RT-qPCR analysis PUM1: Pumilio RNA Binding Family Member 1, HPRT1: Hypoxanthine Phosphoribosyltransferase 1, MRPL19: Mitochondrial Ribosomal Protein L19, (d)Cq: (delta) quantification cycle, TAA: Tumor-associated antigen.


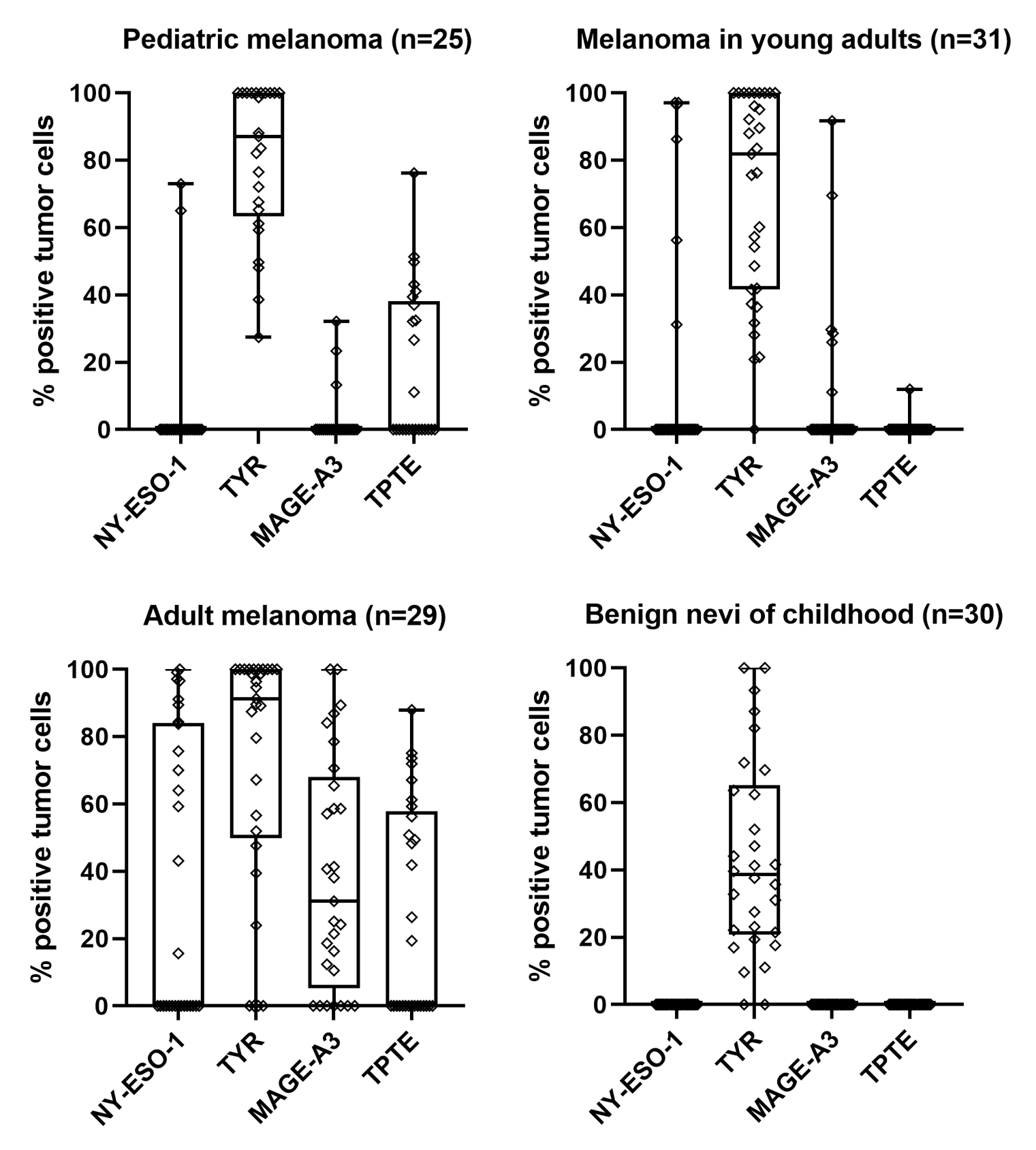


Supplementary Figure 4 Distribution of stained tumor cells (IHC) per marker and cohort Scatter box plots display positively stained tumor cells. Each dot represents an individual sample. Boxes indicate quartiles with median at middle, whiskers drawn to the minimum and up to the maximum value, all points are shown. Staining ≥1% of tumor cells is regarded as positive.

# Supplementary Tables

Supplementary Table 1 Oligonucleotide sequences used in RT-qPCR

| **Oligonucleotide name** | **Sequence** |
| --- | --- |
| TYR forward | CTGCCAACGATCCTATCTTCC |
| TYR reverse | CCATGTAGGATTCCCGGTTATG |
| TYR probe | AGTTTATCCAGAAGCCAATGCACCCA |
| NY-ESO-1 forward | CGTGCCAGGGGTGCTTCT |
| NY-ESO-1 reverse | GCAGCAGTCAGTCGGATAGTCAG |
| NY-ESO-1 probe | TTCACTGTGTCCGGCAACATACTGA |
| TPTE forward | TCAGAAAACAAAAGGCGATACAC |
| TPTE reverse | GAAACCGCACAACTTCCTTG |
| TPTE probe | AGGGATGGATTTGACCTAGACCTCACT |
| MAGE-A3 forward | CGGTGAGGAGGCAAGGTTC |
| MAGE-A3 reverse | AATGGAGACCCACTGGCAGAT |
| MAGE-A3 forward | CCCGGAGGAGCACTGAAGGAGA |
| PUM1 forward | TTATTCAGGCACGCAGGTAC |
| PUM1 reverse | CGCATTAGGTCTTTGGAACAG |
| PUM1 probe | AGCAGCAACTGTGGGACTTTTTGAC |
| HPRT1 forward | AGATGGTCAAGGTCGCAAG |
| HPRT1 reverse | GTATTCATTATAGTCAAGGGCATATCC |
| HPRT1 probe | TGGTGAAAAGGACCCCACGAAGT |
| MRPL19 forward | GGAAGAGGACTTGGAGCTACTTT |
| MRPL19 reverse | AATCTCCTGGACCCGAGGATTA |
| MRPL19 probe | TCGAAGGACAAGGTGTCGAGATTTG |

Supplementary Table 2 Pediatric melanoma patient characteristics and TAA status

| **Patient No./Sex** | **Age**  **(y)** | **Depth (mm)** | **MM Type** | **Mutational Status** | **CNVs (n)** | **FU (d) and Outcome** | **Tumor stage at diagnosis**  **(AJCC 2017)** | **TAA count**  **(IHC)** | **NY-ESO-1**  **(IHC)** | **TYR**  **(IHC)** | **MAGE-A3**  **(IHC)** | **TPTE**  **(IHC)** | **NY-ESO-1**  **(RT-qPCR)** | **TYR**  **(RT-qPCR)** | **MAGE-A3**  **(RT-qPCR)** | **TPTE**  **(RT-qPCR)** |
| --- | --- | --- | --- | --- | --- | --- | --- | --- | --- | --- | --- | --- | --- | --- | --- | --- |
| K1/F | 4 | 3.30 | SM | wt | n.a. | 376/A | II | 3 | - | + | + | + | - | + | + | - |
| K2/F | 8 | 0.95 | SSM | OT | n.a. | 125/A | I | 3 | + | + | - | + | + | + | - | + |
| K4/F | 10 | 3.20 | NM | wt | n.a. | 806/A | II | 1 | - | + | - | - | - | + | - | - |
| K5/M | 13 | 5.00 | MET | BRAF | n.a. | 358/D | IV | 3 | + | + | - | + | + | + | - | + |
| K6/F | 15 | 2.00 | SM | wt | n.a. | 307/A | III | 2 | - | + | - | + | - | + | - | - |
| K7/M | 13 | n.a. | OT | GNAQ | n.a. | 594/A | n.a. | 1 | - | + | - | - | - | + | - | - |
| K9/M | 9 | 1.00 | SSM | n.a. | n.a. | 1694/A | I | 1 | - | + | - | - | - | + | - | - |
| K10/M | 11 | 3.00 | SM | wt | 4 | 1025/A | II | 2 | - | + | - | + | - | + | - | - |
| K11/F | 12 | 5.00 | SM | wt | 7 | n.a./A | II | 1 | - | + | - | - | - | + | - | - |
| K12/F | 7 | 2.50 | SM | wt | 10 | n.a./A | II | 2 | - | + | - | + | - | + | - | - |
| K13/F | 5 | 0.95 | SM | wt | 3 | 1017/A | I | 1 | - | + | - | - | - | + | - | - |
| K14/F | 1 | 2.50 | SM | wt | 8 | n.a./A | II | 3 | - | + | + | + | - | + | + | - |
| K15/F | 17 | 4.60 | SSM | TERTp | n.a. | 1097/A | II | 2 | - | + | - | + | - | + | - | - |
| K16/M | 10 | 7.60 | OT | wt | n.a. | 128/A | IV | 3 | - | + | + | + | - | + | + | - |
| K17/M | 11 | 4.20 | SM | wt | 11 | 1425/A | II | 1 | - | + | - | - | - | + | - | - |
| K18/M | 8 | 2.30 | SM | wt | 1 | 2516/A | III | 1 | - | + | - | - | - | + | - | - |
| K19/F | 10 | 0.30 | ALM | wt | n.a. | 1717/A | I | 1 | - | + | - | - | - | + | - | - |
| K20/M | 17 | 2.50 | CNM | BRAF | 6 | 3010/A | II | 1 | - | + | - | - | - | + | - | - |
| K21/M | 9 | 3.50 | SM | wt | 4 | 1550/A | II | 1 | - | + | - | - | - | + | - | - |
| K22/F | 13 | 5.50 | MET | wt | 2 | 1467/A | III | 2 | - | + | - | + | - | + | - | - |
| K23/F | 3 | 11.20 | SM | wt | n.a. | 318/A | II | 2 | - | + | - | + | - | + | - | - |
| K24/M | 10 | 5.00 | SSM | BRAF | n.a. | 2191/D | III | 1 | - | + | - | - | - | + | - | - |
| K27/M | 15 | 14.00 | CNM | NRAS | n.a. | 2480/D | II | 1 | - | + | - | - | - | + | - | - |
| K29/F | 2 | 8.00 | CNM | wt | 11 | 76/D | III | 1 | - | + | - | - | - | + | - | - |
| K30/M | 4 | 2.00 | SM | wt | 9 | 3655/A | I | 1 | - | + | - | - | - | + | - | - |

Abbreviations: A, alive. ALM, Acral lentiginous melanoma. CNM, congenital nevus melanoma. CNVs, copy number variations. D, died of melanoma. d, days. F, female. FU, follow-up. IHC, immunohistochemistry. M, male. MET, metastasis. MM, malignant melanoma. n.a. not available. NM, nodular melanoma. OT, other type of melanoma. RT-qPCR, reverse transcription quantitative real-time polymerase chain reaction. SM, spitzoid melanoma. SSM, superficial spreading melanoma. TAA, tumor-associated antigen. TERTp, TERT promoter mutation. wt, wildtype. y, years.

Supplementary Table 3 Melanoma in young adults characteristics and TAA status

| **Patient No./Sex** | **Age**  **(y)** | **Depth (mm)** | **MM Type** | **FU (d) and Outcome** | **Tumor stage at diagnosis**  **(AJCC 2017)** | **TAA count** | **NY-ESO-1**  **(IHC)** | TYR  **(IHC)** | **MAGE-A3**  **(IHC)** | **TPTE**  **(IHC)** | **NY-ESO-1 (RT-qPCR)** | **TYR**  **(RT-qPCR)** | **MAGE-A3**  **(RT-qPCR)** | **TPTE**  **(RT-qPCR)** |
| --- | --- | --- | --- | --- | --- | --- | --- | --- | --- | --- | --- | --- | --- | --- |
| J1/M | 30 | 0.53 | SSM | 1920/A | I | 2 | - | + | + | - | - | + | + | - |
| J2/F | 28 | 0.52 | SM | 46/A | I | 1 | - | + | - | - | - | + | - | - |
| J3/F | 25 | 0.42 | SSM | 1341/A | I | 1 | - | + | - | - | invalid | invalid | invalid | invalid |
| J4/F | 25 | 2.50 | NM | 2355/A | III | 2 | + | + | - | - | + | + | - | - |
| J5/M | 29 | 6.50 | SM | 2037/A | II | 1 | - | + | - | - | - | + | - | - |
| J6/M | 26 | 0.40 | SSM | 68/A | I | 2 | - | + | - | + | - | + | - | + |
| J7/F | 26 | 0.25 | SSM | 1407/A | I | 1 | - | + | - | - | - | + | - | - |
| J8/F | 27 | 0.30 | SSM | 1604/A | I | 1 | - | + | - | - | - | + | - | - |
| J9/M | 21 | 0.30 | SSM | 1561/A | I | 1 | - | + | - | - | - | + | - | - |
| J10/F | 29 | 2.50 | SM | n.a./A | II | 1 | - | + | - | - | - | + | - | - |
| J11/M | 30 | 0.30 | SSM | 76/A | I | 2 | + | + | - | - | + | + | - | - |
| J12/M | 30 | 0.75 | SSM | 434/A | I | 1 | - | + | - | - | - | - | - | - |
| J13/F | 20 | 0.45 | SSM | 2416/A | I | 2 | + | + | - | - | + | + | + | - |
| J14/F | 27 | 0.46 | SSM | 998/A | I | 3 | + | + | + | - | + | + | + | - |
| J15/F | 27 | 0.45 | SSM | 592/A | I | 1 | - | + | - | - | - | + | - | - |
| J16/F | 25 | 0.41 | SSM | 412/A | I | 1 | - | + | - | - | - | - | - | - |
| J18/M | 21 | 14.00 | NM | 1623/D | III | 2 | - | + | + | - | - | + | + | - |
| J19/F | 30 | 0.60 | SSM | 685/A | I | 3 | + | + | + | - | + | + | + | - |
| J20/F | 23 | 0.80 | SSM | 1622/A | I | 1 | - | + | - | - | - | + | - | - |
| J21/F | 27 | 2.80 | SM | n.a./A | II | 2 | + | + | - | - | + | + | - | - |
| J22/F | 27 | 0.45 | SSM | 1292/A | I | 0 | - | - | - | - | - | - | - | - |
| J23/M | 25 | 3.50 | SSM | 3796/A | II | 1 | - | + | - | - | - | + | - | - |
| J24/M | 26 | 1.10 | SSM | 1237/A | I | 1 | - | + | - | - | - | + | - | - |
| J25/F | 21 | 1.25 | SSM | 3658/A | I | 1 | - | + | - | - | - | + | - | - |
| J26/M | 20 | 3.30 | NM | 2514/A | II | 1 | - | + | - | - | - | + | - | - |
| J27/F | 28 | 0.75 | SSM | 1560/A | I | 1 | - | + | - | - | - | + | - | - |
| J28/F | 26 | 2.00 | SSM | 762/A | I | 2 | - | + | + | - | - | + | + | - |
| J29/M | 30 | 1.37 | SSM | 3428/A | I | 1 | - | + | - | - | - | + | - | - |
| J30/M | 25 | 0.85 | ALM | 1008/A | I | 2 | - | + | + | - | - | + | + | - |
| J31/M | 22 | 1.00 | ALM | 1398/A | I | 1 | - | + | - | - | - | + | + | - |
| J32/F | 21 | 1.10 | SSM | 1386/A | I | 1 | - | + | - | - |  |  |  |  |

Abbreviations: A, alive. ALM, Acral lentiginous melanoma. D, died of melanoma. d, days. F, female. FU, follow-up. IHC, immunohistochemistry. M, male. MM, malignant melanoma. n.a. not available. NM, nodular melanoma. RT-qPCR, reverse transcription quantitative real-time polymerase chain reaction. SM, spitzoid melanoma. SSM, superficial spreading melanoma. TAA, tumor-associated antigen. y, years.

Supplementary Table 4 Adult melanoma characteristics and TAA status

| **Patient No./Sex** | **Age (y)** | **Depth (mm)** | **MM Type** | **FU (d) and Outcome** | **Tumor stage at diagnosis (AJCC 2017)** | **TAA count** | **NY-ESO-1 (IHC)** | **TYR**  **(IHC)** | **MAGE-A3**  **(IHC)** | **TPTE (IHC)** | **NY-ESO-1 (RT-qPCR)** | **TYR**  **(RT-qPCR)** | **MAGE-A3**  **(RT-qPCR)** | **TPTE**  **(RT-qPCR)** |
| --- | --- | --- | --- | --- | --- | --- | --- | --- | --- | --- | --- | --- | --- | --- |
| **A1/M** | 66 | 3.30 | SSM | 252/A | III | 4 | + | + | + | + | + | + | + | + |
| A2/F | 76 | 0.95 | SSM | 235/A | I | 0 | - | - | - | - | - | - | - | - |
| A3/M | 69 | 4.00 | NM | 66/A | II | 3 | + | + | + | - | + | + | + | - |
| A4/F | 61 | 3.22 | SSM | 2485/A | II | 3 | + | + | + | - | - | + | + | - |
| A5/M | 64 | n.a. | MET | 305/A | IV | 4 | + | + | + | + | + | + | + | + |
| A6/M | 73 | 2.00 | LMM | 195/A | I | 4 | + | + | + | + | + | + | + | + |
| A8/F | 41 | 0.30 | SSM | 1692/A | I | 1 | - | - | + | - | - | - | + | - |
| A9/M | 67 | 1.00 | SSM | 75/A | I | 3 | + | + | + | - | - | + | - | - |
| A10/F | 60 | 3.00 | NM | 1922/A | II | 2 | + | + | - | - | + | + | - | - |
| A11/M | 33 | 5.00 | SM | 1775/A | II | 3 | + | + | - | + | - | + | - | + |
| A12/M | 75 | 2.50 | SSM | 488/A | II | 3 | + | + | + | - | + | + | + | - |
| A13/F | 36 | 0.95 | SSM | 1112/A | I | 1 | - | + | - | - | - | + | - | - |
| A14/F | 47 | 2.50 | SSM | 1188/A | II | 2 | - | + | + | - | - | + | - | - |
| A15/F | 73 | 4.50 | ALM | 608/D | III | 4 | + | + | + | + | + | - | + | + |
| A16/F | 83 | 7.50 | SSM | 320/A | II | 4 | + | + | + | + | + | + | + | + |
| A17/M | 43 | 4.25 | SSM | 521/D | III | 3 | - | + | + | + | - | + | + | + |
| A18/F | 49 | 2.30 | SSM | 2605/A | III | 4 | + | + | + | + | + | + | + | + |
| A19/F | 70 | 0.30 | SSM | 1595/A | I | 1 | - | + | - | - | - | + | - | - |
| A20/F | 81 | 2.50 | SSM | 400/D | II | 1 | - | - | + | - | - | - | + | - |
| A21/F | 70 | 3.50 | ALM | 94/A | III | 1 | - | + | - | - | - | + | - | - |
| A22/M | 68 | n.a. | MET | 621/D | IV | 3 | - | + | + | + | - | + | + | + |
| A23/M | 89 | 7.00 | SSM | 182/D | III | 3 | - | + | + | + | - | + | + | + |
| A24/F | 77 | 5.00 | SSM | 213/A | III | 3 | + | + | + | - | + | + | + | - |
| A25/M | 37 | 1.70 | SSM | 197/A | II | 2 | - | + | + | - | - | + | + | - |
| A26/M | 77 | 0.30 | SSM | 240/A | I | 1 | - | + | - | - | - | + | - | - |
| A27/M | 95 | 14.00 | OT | 43/D | III | 2 | - | - | + | + | - | - | + | + |
| A28/F | 42 | 1.50 | SSM | 1988/A | I | 4 | + | + | + | + | + | + | + | + |
| A29/F | 92 | 8.00 | LMM | 134/A | III | 3 | - | + | + | + | - | + | + | + |
| A30/F | 47 | 2.00 | SSM | 2258/A | I | 3 | - | + | + | + | - | + | + | + |

Abbreviations: A, alive. ALM, Acral lentiginous melanoma. D, died of melanoma. d, days. F, female. FU, follow-up. IHC, immunohistochemistry. LMM, lentigo maligna melanoma. M, male. MET, metastasis. MM, malignant melanoma. n.a. not available. NM, nodular melanoma. OT, other type of melanoma. RT-qPCR, reverse transcription quantitative real-time polymerase chain reaction. SM, spitzoid melanoma. SSM, superficial spreading melanoma. TAA, tumor-associated antigen. y, years.

**Supplementary Table 5 Benign melanocytic nevi of childhood characteristics and TAA status**

| **Patient No./Sex** | **Age (y)** | **TAA count (IHC)** | **NY-ESO-1 (IHC)** | **TYR**  **(IHC)** | **MAGE-A3 (IHC)** | **TPTE (IHC)** | **NY-ESO-1 (RT-qPCR)** | **TYR**  **(RT-qPCR)** | **MAGE-A3 (RT-qPCR)** | **TPTE**  **(RT-qPCR)** |
| --- | --- | --- | --- | --- | --- | --- | --- | --- | --- | --- |
| **N1/F** | 14 | 1 | - | + | - | - | - | + | - | - |
| N2/F | 7 | 1 | - | + | - | - | - | + | - | - |
| N3/M | 4 | 1 | - | + | - | - | - | + | - | - |
| N4/F | 13 | 1 | - | + | - | - | - | + | - | - |
| N5/F | 13 | 0 | - | - | - | - | - | - | - | - |
| N6/M | 7 | 1 | - | + | - | - | - | + | - | - |
| N7/M | 12 | 1 | - | + | - | - | - | + | - | - |
| N8/M | 14 | 1 | - | + | - | - | - | + | - | - |
| N9/M | 17 | 1 | - | + | - | - | - | + | - | - |
| N10/M | 15 | 1 | - | + | - | - | - | + | - | - |
| N11/M | 7 | 1 | - | + | - | - | - | + | - | - |
| N12/M | 12 | 1 | - | + | - | - | - | + | - | - |
| N13/F | 13 | 1 | - | + | - | - | - | + | - | - |
| N14/F | 13 | 1 | - | + | - | - | - | + | - | - |
| N15/F | 15 | 1 | - | + | - | - | - | + | - | - |
| N16/M | 16 | 1 | - | + | - | - | - | + | - | - |
| N17/F | 0 | 1 | - | + | - | - | - | + | - | - |
| N18/M | 16 | 1 | - | + | - | - | - | + | - | - |
| N19/F | 16 | 1 | - | + | - | - | - | + | - | - |
| N20/M | 6 | 1 | - | + | - | - | - | + | - | - |
| N21/F | 1 | 1 | - | + | - | - | - | + | - | + |
| N22/M | 1 | 1 | - | + | - | - | - | + | - | - |
| N23/M | 6 | 1 | - | + | - | - | - | + | - | - |
| N24/F | 0 | 1 | - | + | - | - | - | + | - | - |
| N25/F | 10 | 0 | - | - | - | - | - | - | - | - |
| N26/F | 12 | 1 | - | + | - | - | - | + | - | - |
| N27/F | 12 | 1 | - | + | - | - | - | + | - | - |
| N28/M | 3 | 1 | - | + | - | - | - | + | - | - |
| N29/M | 13 | 1 | - | + | - | - | - | + | - | - |
| N30/F | 7 | 1 | - | + | - | - | - | + | - | - |

Abbreviations: F, female. IHC, immunohistochemistry. M, male. RT-qPCR, reverse transcription quantitative real-time polymerase chain reaction. TAA, tumor-associated antigen. y, years.
